# Eosinophilic esophagitis

**DOI:** 10.1186/s13223-024-00929-0

**Published:** 2024-12-19

**Authors:** Stephanie C. Erdle, Stuart Carr, Edmond S. Chan, Kara Robertson, Wade Watson

**Affiliations:** 1 Division of Allergy, Department of Pediatrics, University of British Columbia, BC Children’s Hospital, Vancouver, BC Canada; 2 Snö Asthma & Allergy, Abu Dhabi, United Arab Emirates; 3 Division of Allergy & Immunology, Department of Internal Medicine, Western University, London, ON Canada; 4 Division of Allergy, Department of Pediatrics, Dalhousie University, IWK Health Centre, Halifax, NS Canada

**Keywords:** Eosinophilic esophagitis, Diagnosis, Treatment, Prognosis, Elemental diet, Empiric dietary restrictions, Proton pump inhibitors, Endoscopic dilation

## Abstract

Eosinophilic esophagitis (EoE) is an atopic condition of the esophagus that has become increasingly recognized. Diagnosis of the disorder is dependent on the patient’s clinical manifestations and must be confirmed by histologic findings on esophageal mucosal biopsies. The epidemiology, pathophysiology, diagnosis, treatment, and prognosis of EoE are discussed in this review.

## Key take-home messages

• EoE is an atopic condition of the esophagus that has become increasingly recognized.
• Endoscopic mucosal biopsy revealing *≥* 15 eosinophils/HPF in one or more specimens remains the most important diagnostic test for EoE, and is mandatory for diagnosis.
• Patients with EoE should be referred to an allergist to help optimize treatment, and manage concurrent atopic conditions.
• Skin or specific IgE blood testing for foods is not indicated for the identification of EoE triggers, as this is not an IgE-mediated disease.
• A trial of PPI is no longer a diagnostic criterion for EoE. To simplify, there is no longer a need to consider the term PPI-REE clinically.
• The elemental diet and empiric dietary restrictions are associated with high rates of clinical and histologic improvement in patients with EoE.
• Pharmacologic management options include PPI, topical corticosteroids delivered to the esophagus or biologics.
• Esophageal endoscopic dilation is most commonly used in adults with established esophageal strictures.
• While it is unclear whether OIT causes or unmasks EoE, continuing or initiating OIT as long as EoE is well controlled could be considered. More research is needed.

## Introduction

Eosinophilic esophagitis (EoE) is an atopic inflammatory disease of the esophagus that has become increasingly recognized in children and adults.

Eosinophils are typically present throughout the gastrointestinal tract since it is continuously exposed to foods, environmental allergens, toxins, and pathogens. Interestingly, in healthy individuals, the esophagus is unique because eosinophils are generally absent. In EoE, however, eosinophils infiltrate the esophagus, contributing to tissue damage and chronic inflammation. EoE is defined as a clinicopathologic disorder characterized by symptoms of esophageal dysfunction and the presence of *≥* 15 eosinophils per high power field (HPF) in one or more esophageal biopsy specimens, in the absence of other non-EoE disorders which can cause or contribute to esophageal eosinophilia [1–3].

The increasing number of recognized cases of EoE has resulted in a dramatic expansion of the medical literature surrounding the disease. This article provides a practical overview of recent literature surrounding the epidemiology, pathophysiology, diagnosis, treatment, and prognosis of EoE.

## Epidemiology

Current prevalence estimates of EoE in North America and Europe range from 2.3 to 90.7 per 100,000 persons [4–6], and the literature suggests that the prevalence is increasing [2, 4, 5]. The reasons for this increase are poorly understood, and there is debate as to whether the new cases of EoE being diagnosed represent a true increase in prevalence or, rather, increased recognition of latent disease.

There are ethnic and gender variations in the prevalence of EoE, with the majority of cases reported in Caucasian males [4, 7]. EoE is predominant in socioeconomically developed countries but has the highest prevalence in the United States, Western Europe, and Australia, compared with Japan and China [4]. Evidence for ethnic variation is further supported by a Canadian study which found a paucity of East Asian (including Chinese and Japanese) pediatric patients, compared with white and South Asian patients, in the EoE cohort [8]. Although few studies have examined EoE in the African American population, a recent genome-wide association study identified three significant loci associated with EoE in African American patients [9].

## Risk factors

In addition to gender (male predominance) and race (mainly a disease of Caucasian individuals), established risk factors for EoE include atopy and other allergic conditions (e.g., allergic rhinitis, elevated serum immunoglobulin E [IgE] to common aeroallergens, asthma, atopic dermatitis and pollen food allergy syndrome). Patients with concomitant EoE and seasonal allergic rhinitis may have more EoE exacerbations during peak pollen seasons [10, 11].

Other recognized genetic and environmental risk factors for EoE include: alterations in gut barrier function (e.g., from gastroesophageal reflux disease [GERD]); variation in the nature and timing of oral antigen exposure (e.g., secondary to infant feeding practices, proton pump inhibitor [PPI] use and commercial food processing); variation in the nature and timing of aeroallergen exposure (seasonal, geographic and secondary to migration); lack of early exposure to microbes and an altered microbiome (e.g., from caesarean section or lack of breastfeeding) and factors relating to fibrous remodeling [12–15].

## Pathophysiology

EoE likely results from an interplay of genetic, immune system and environmental factors as well as mechanisms of mucosal damage and fibrosis [16, 17]. Evidence suggests that the disease is associated with type 2 inflammation, which is typical of other atopic conditions. In the case of EoE, this type 2 inflammation is antigen-driven, with both foods and environmental allergens implicated. Elevated levels of the type 2 inflammatory cytokines interleukin (IL)-4, IL-5, and IL-13, as well as mast cells, have been found in the esophageal biopsies of EoE patients [12, 13, 16, 17]. These cytokines play an important role in the activation and recruitment of eosinophils to the esophagus. Eosinophils, in turn, play an integral role in the remodeling of esophageal tissues, which is observed histologically as subepithelial fibrosis. Eosinophils contribute to fibrosis through degranulation and secretion of their granule cationic proteins, particularly major basic protein (MBP), and elaboration of fibrogenic growth factors such as transforming growth factor-beta (TGF-β) [16].

The male predominance of EoE, as well as family history, twin concordance and genome-wide association studies, suggest that there is a genetic predisposition to EoE [12, 16–18]. The gene for eotaxin-3 (a chemokine involved in promoting eosinophil accumulation and adhesion) is overexpressed in patients with EoE [19]. Similarly, the expression of genes involved in epidermal differentiation has been found to be markedly decreased in EoE, suggesting barrier disruption may play a role [20]. Genome-wide association studies have identified specific loci associated with EoE [18, 21–25].

EoE is thought to represent a predominantly non-IgE-mediated allergic response to food and environmental allergens [17, 26, 27]. Although many patients with EoE have positive skin prick tests (which detect IgE-mediated responses) to foods and/or environmental allergens, these tests do not accurately identify causative foods in EoE patients [28, 29]. Rather, these findings are most likely reflective of other comorbid atopic conditions, which occur more frequently in patients with EoE [11, 30].

More recently, EoE has been linked to a small percentage of patients undergoing oral immunotherapy (OIT) (see Oral Immunotherapy article in this supplement) for the treatment of IgE-mediated food allergy. It remains unclear whether the OIT causes the EoE in all patients, or rather “unmasks” it in some patients who had pre-existing, undiagnosed esophageal eosinophilia [31–36]. A recent Canadian publication proposed a practical guide to managing gastrointestinal symptoms in patients undergoing OIT [37]. In general, OIT can be initiated or continued in patients with EoE as long as the disorder is well controlled. Additional studies are required to further evaluate the relationship between OIT and EoE.

EoE has also been reported during sublingual immunotherapy (SLIT) to aeroallergens. This has been largely limited to case reports, with the majority experiencing improvement in symptoms after SLIT discontinuation, PPI use, or switching from a swallow method to spit method after the period of absorption [38, 39].

## Diagnosis and investigations

Since the physical examination of patients with EoE is often unrevealing, the diagnosis of EoE is dependent on the patient’s clinical manifestations, endoscopic assessment of the esophagus and histologic findings on esophageal mucosal biopsies.

### Clinical manifestations

Although the typical onset of EoE is in childhood, the disease can be found in all age groups, and symptoms vary depending on the age of presentation [30, 40] (see Table 1 for a summary of the clinical manifestations of EoE). Clinical manifestations in infants and toddlers generally include vomiting, food refusal, choking with meals and, less commonly, failure to thrive. Cardinal symptoms in school-aged children and adolescents include dysphagia (difficulty swallowing), food impaction, and choking/gagging with meals, particularly while eating foods with coarse textures. In this patient population, less severe symptoms may precede these prominent symptoms, including abdominal/chest pain, vomiting, regurgitation, heartburn and reflux symptoms. A careful history in children and adolescents with EoE reveals that they have learned to compensate for these symptoms by eating slowly, chewing excessively or taking small bites, drinking excessively with meals, lubricating meals inordinately with sauces, and avoiding specific food consistencies such as meats (or other foods with coarse textures) [41, 42].

**Table 1:** Clinical manifestations of EoE

	Infants/Toddlers	Children	Adults
**Symptoms**	• Feeding aversion/intolerance • Vomiting • Food refusal • Choking with meals • Failure to thrive • Sleep disturbance	• Dysphagia • Choking/gagging with coarse textures • Food impactions • Abdominal/chest pain • Throat pain • Vomiting/regurgitation • Nausea • Sleep disturbance • Decreased appetite	• Dysphagia (predominant) • Food impactions • Food avoidance • Intractable heartburn • Regurgitation • Retrosternal pain • Chest pain
**Associated conditions**	• Food allergy • Atopic dermatitis	• Asthma • Allergic rhinitis • Food allergy	• Asthma • Allergic rhinitis • Pollen food allergy syndrome

The predominant symptom in adults is dysphagia; however, intractable heartburn and food avoidance may also be present. Due to the long-standing inflammation and possible resultant scarring that has gone unrecognized, adults presenting with EoE tend to have more episodes of esophageal food impaction as well as other esophageal abnormalities such as Schatzki ring (a narrow ring of tissue located just above the junction of the esophagus and stomach), esophageal webs (small, thin growths of tissue that partially block the esophagus) and, in some cases, achalasia (an esophageal motility disorder characterized by difficulty swallowing and regurgitation). However, it is important to note that some patients with EoE are asymptomatic, and suspicion of the disease is based upon incidental findings at endoscopy that is performed for other indications or upon evidence of food impaction.

Many symptoms of EoE overlap with GERD, however up to 75% of patients with EoE have a personal or family history of atopic disease (e.g., asthma, eczema, allergic rhinitis, pollen food allergy syndrome and/or food allergies) [11, 30]. It is important to note that up to one-half of patients who meet the diagnostic criteria for EoE will respond to PPI monotherapy, and until recently, this phenomenon was referred to as PPI-responsive esophageal eosinophilia (PPI-REE) [2], and was viewed as a distinct clinical disorder, albeit with some controversy [43–47]. More recent evidence confirms that the ribonucleic acid (RNA) expression profiles are similar for patients with classic EoE and those with PPI-REE, and distinct from those with GERD [48, 49]. As a result, consensus diagnostic criteria indicate that EoE and PPI-REE are on the same spectrum (i.e., there is no longer the need to use the term PPI-REE clinically), and that PPI could be considered a treatment for EoE [50].

### Endoscopy

**Fig. 1:**
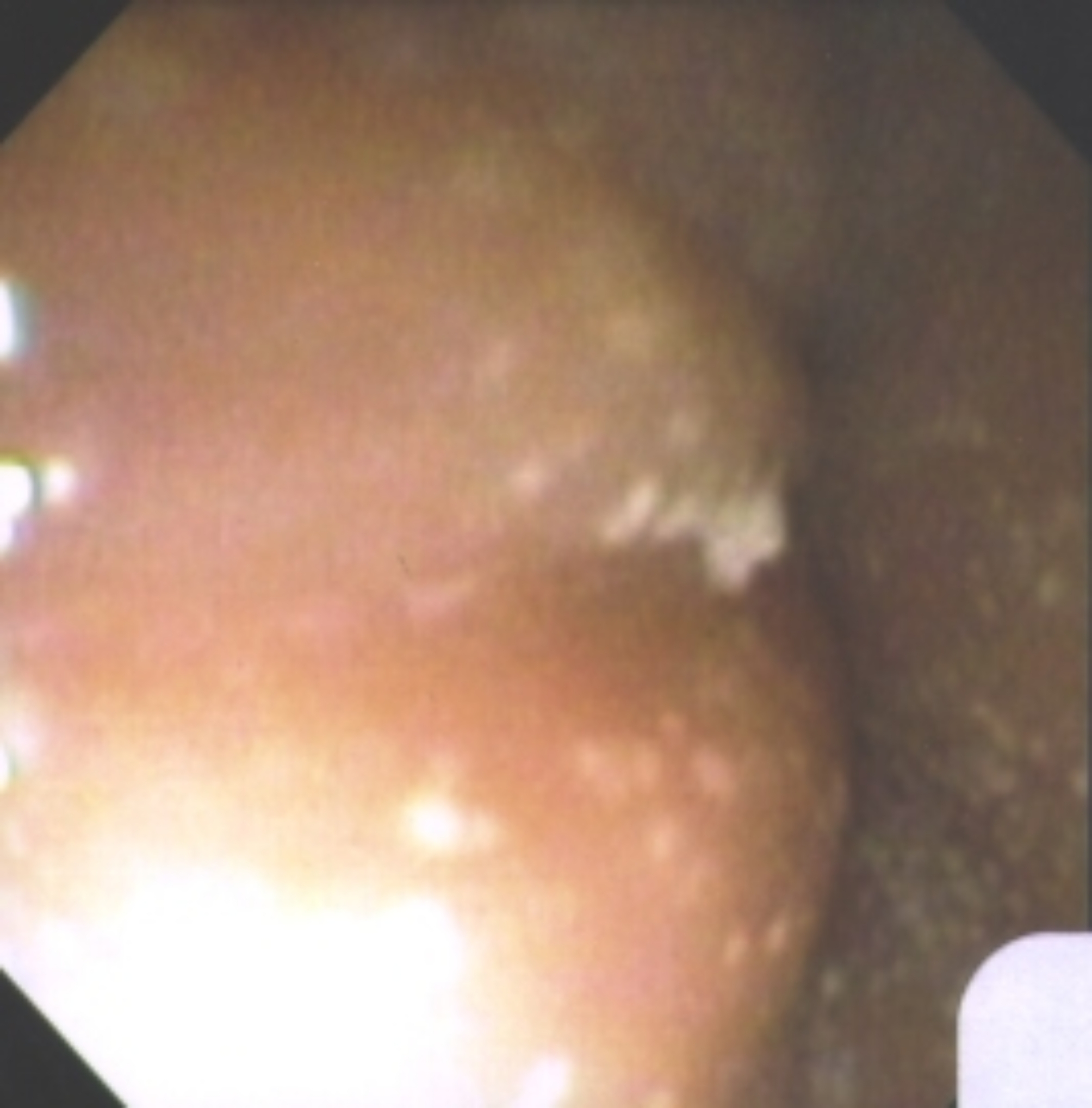
Endoscopic features of EoE: White exudates

**Fig. 2:**
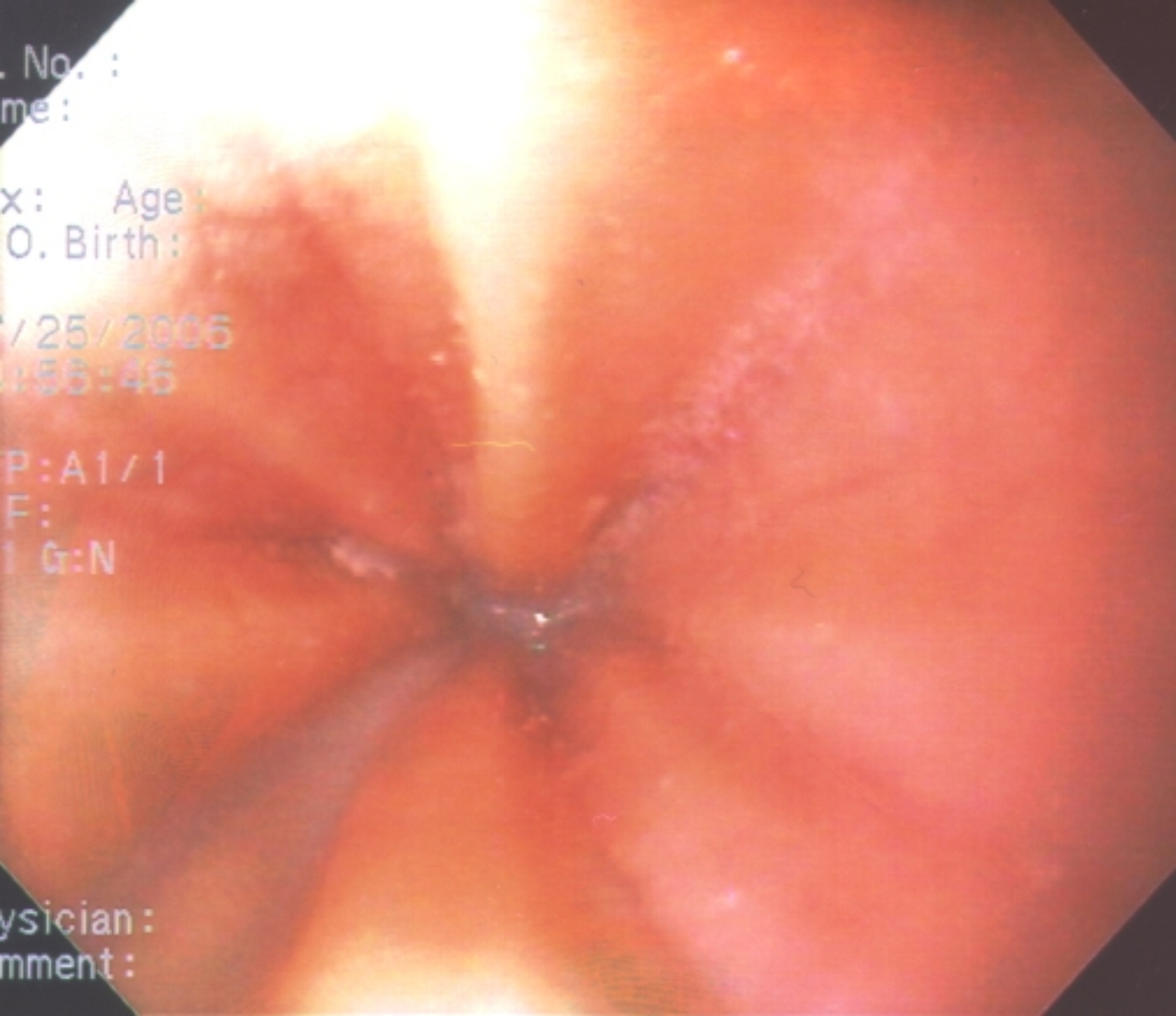
Endoscopic features of EoE: Linear Furrows

**Fig. 3:**
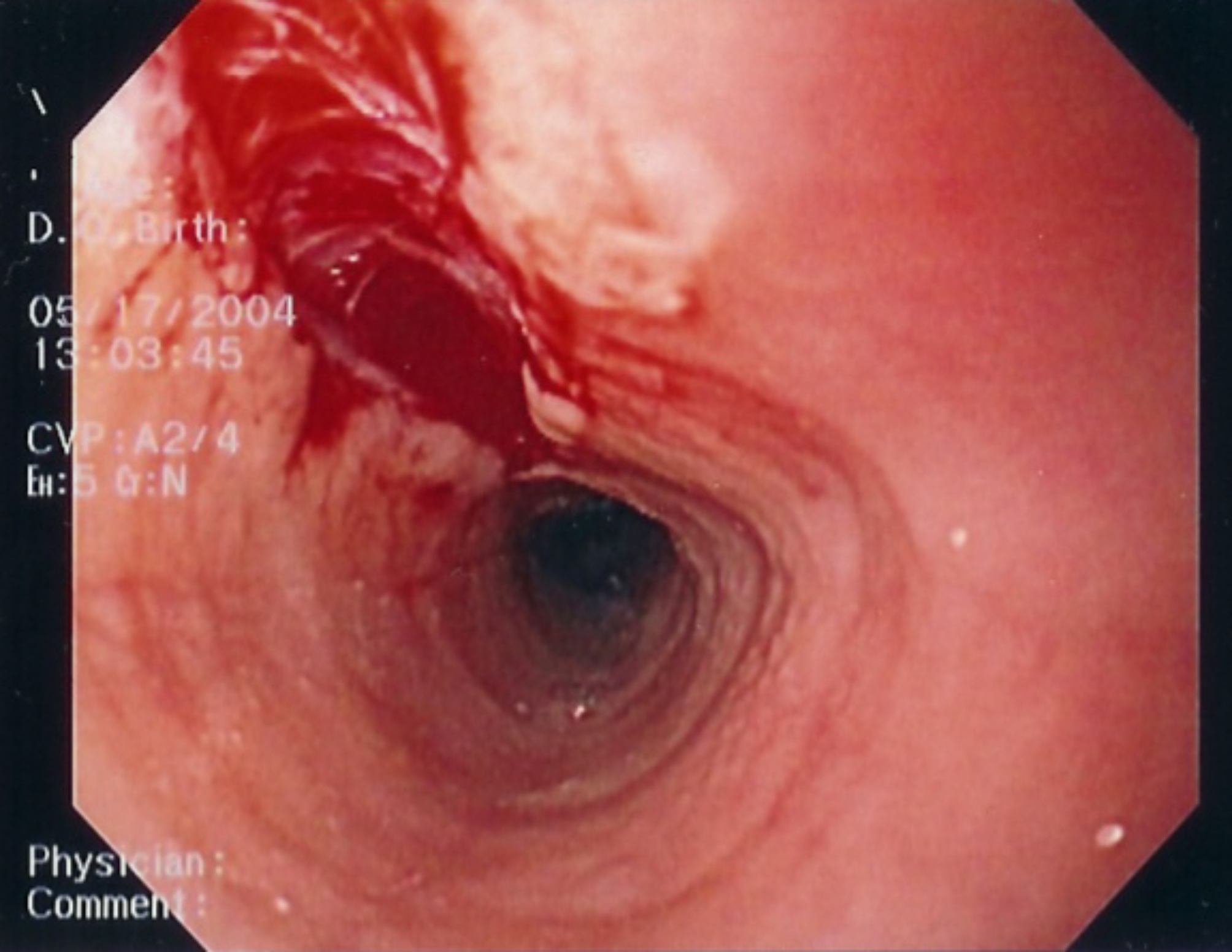
Endoscopic features of EoE: Linear tear plus concentric rings

**Fig. 4:**
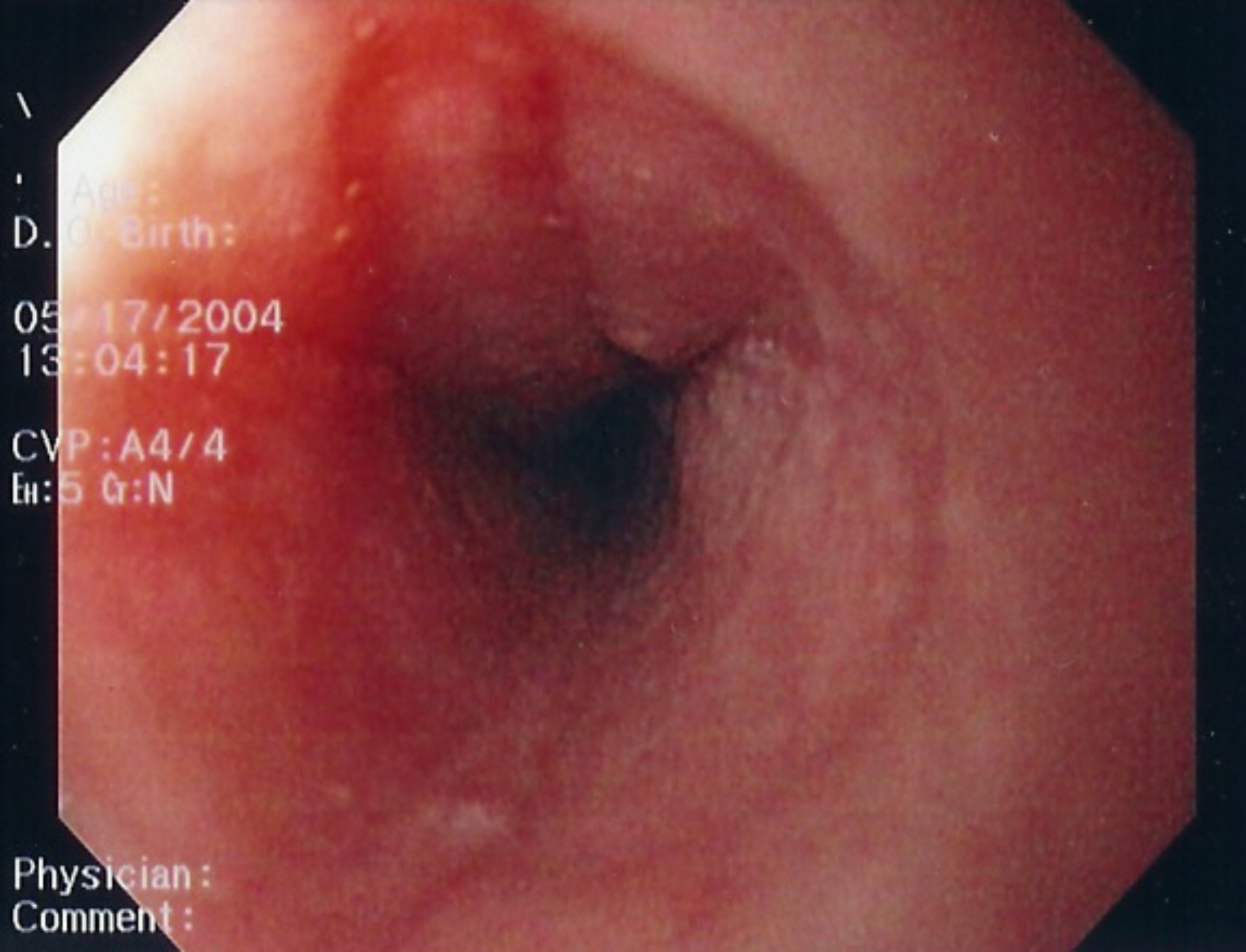
Endoscopic features of EoE: Edema, furrows, exudate

Endoscopic features of EoE have been well-characterized and include linear furrowing (ridges or furrows in the esophageal wall), concentric rings, white speckled exudates (eosinophilic abscesses), Schatzki ring, small-calibre esophagus, and linear superficial mucosal tears that occur after introduction of the endoscope [2]. Table 2 provides a more detailed description of each of these features. Images of exudates, linear furrows and tears are provided in Figs. 1, 2, 3 and 4. Note that, prior to endoscopy, a barium swallow may be considered in severely symptomatic patients to rule out severe small-calibre esophagus.

**Table 2:** Endoscopic features of EoE

Endoscopic feature	Description
Linear furrowing	• Vertical esophageal lines or ridges in the esophageal wall
Concentric rings	• Multiple rings that may be fine, web-like or thickened (also termed the “corrugated” or “ringed” esophagus)
White speckled exudates	• Patches of whitish papules (1–2 mm in diameter) • Resembles esophageal candidiasis
Schatzki ring	• Narrow ring of tissue located just above the junction of the esophagus and stomach
Small-calibre esophagus	• Narrowed esophagus, with fixed internal diameter • Featureless, unchanging column • Poor expansion on air insufflation • Proximal and/or distal stenosis
Linear superficial mucosal tears	• Mucosal abrasions or shearing that occur upon minimal contact (e.g., after simple passage of a routine endoscope)

### Esophageal mucosal biopsies

Currently, endoscopic mucosal biopsy remains the most important diagnostic test for EoE and is required to confirm the diagnosis. Biopsy specimens from both the proximal or mid and distal esophagus should be obtained regardless of the gross appearance of the mucosa, as well as from areas revealing endoscopic abnormalities [1]. At least four biopsies are required to obtain adequate sensitivity for the detection of EoE (5–6 biopsies are generally recommended).

A definitive diagnosis of EoE is based on the presence of at least 15 eosinophils/HPF in the esophageal biopsies of patients with symptoms of esophageal dysfunction. GERD can increase eosinophilic infiltration in the distal esophagus, however, eosinophils associated with GERD generally occur at a lower density (i.e., < 15/HPF).

There have been recent efforts to develop an EoE severity index that can be used both in research and clinically [51].

### Allergy assessment

A referral to an allergist is recommended for optimal management of EoE, although the role of the allergist may vary according to local practice. In some centers, EoE management is directed by gastroenterology, while in others, this may be led by allergy, or collaboratively between gastroenterology and allergy in multidisciplinary clinics. Regardless, a referral to allergy is recommended in all patients with EoE given the high prevalence of comorbid atopic conditions. Current methods of food allergy testing (skin prick testing or serum specific IgE testing), which identify IgE-mediated sensitization, do not identify EoE triggers, and should therefore not be performed to identify food triggers of EoE [29, 52, 53]. Testing may be considered in cases of comorbid IgE-mediated food allergy or for assessment of allergic rhinoconjunctivitis. Physicians should discourage food allergy testing if the patient is eating foods without a history of immediate reactions. Testing may be performed in select cases if there has been a significant period of specific food avoidance in an atopic individual given the risk of loss of tolerance over time, in order to facilitate reintroduction [54–57].

Atopy patch testing has been used in some centres for the potential identification of delayed, non-IgE-mediated reactions, however, it has not been shown to be helpful in identifying food triggers in EoE and is therefore not recommended [58].

## Treatment

**Fig. 5:**
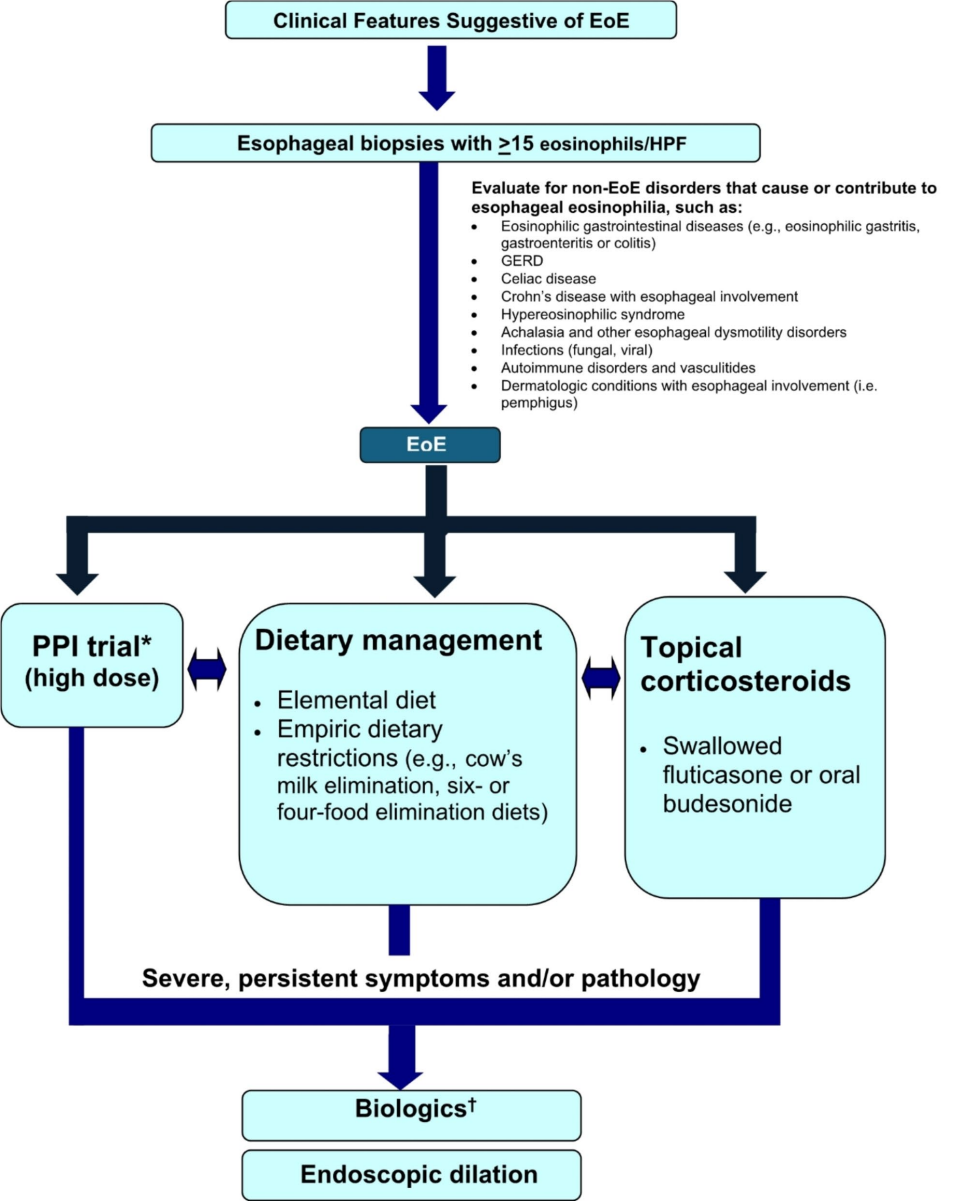
Simplified algorithm for the diagnosis and management of EoE [50, 59, 60] *Some practitioners may desire PPI as initial treatment due to their low cost, convenience, and safety ^†^Dupilumab is the only biologic currently approved for EoE by Health Canada *EoE: eosinophilic esophagitis; GERD: gastroesophageal reflux disease; HPF: high power field; PPI: proton pump inhibitor*

Treatment strategies available for EoE fall into three categories: (1) avoidance of triggers through dietary modification; (2) pharmacologic therapy; and (3) mechanical dilation of the esophagus [59]. A simplified algorithm for the diagnosis and management of EoE is shown in Fig. 5 [50, 59, 60].

### Dietary management

Two effective dietary approaches for the management of EoE have emerged: (1) the elemental diet and (2) empiric food elimination diets (FED; e.g., one FED [1FED] with cow’s milk alone; two FED [2FED – cow’s milk, wheat], four FED [4FED – cow’s milk, egg, wheat, and legumes] or six FED (6FED – cow’s milk, wheat, eggs, soy, peanuts/tree nuts and fish/shellfish]). Targeted dietary elimination (based on results of skin prick testing or patch testing) is not effective and, therefore, not recommended in recent guidelines [59, 61].

#### (a) Elemental diet

The elemental diet involves the removal of all sources of potentially allergenic protein from the patient’s diet through the use of an amino acid-based formula for nutritional support. Assuming there is a favorable clinical and histologic response, one new food per week is reintroduced in a sequential fashion, beginning with the least allergenic foods (fruits and vegetables) to the most highly allergenic (e.g., cow’s milk, wheat, and egg). A repeat endoscopic assessment is recommended after the reintroduction of every 3–5 foods to ensure that the inflammation has not recurred. Although the elemental diet is associated with high rates of clinical and histologic improvement in children with EoE (i.e., > 90%), symptoms often recur after normalization of the patient’s diet [62, 63]. Furthermore, given the unpalatable taste of the formula, most patients require feeding by nasogastric tube which may lead to adherence issues and impaired quality of life (QoL), particularly in adolescents and adults. In this latter population, the elemental diet is only effective in approximately 70% of patients [64].

#### (b) Food elimination diet (FED)

Empiric FED are often employed before considering an elemental diet. Empiric FED involve the elimination of the most common EoE food triggers. Cow’s milk is the most implicated triggering food (74%), followed by wheat (26%) and egg (17%) [65]. Many centres choose 1FED with cow’s milk alone (all dairy products, including other cross-reactive mammalian milk products [e.g., goat, sheep]) as the first trial based on data suggesting a response rate approaching that of the six-food elimination diet (6FED), but with greater convenience/feasibility [65–68]. Recent reports also suggest that there may be a risk of new IgE-mediated allergy development if a food is eliminated from the diet, further supporting the 1FED to avoid unnecessary elimination of food allergens [54–57]. For patients in whom 1FED is insufficient, a “step-up” approach to a 2FED, 4FED or 6FED can be considered.

With all dietary approaches, it remains unclear how long specific foods need to be avoided, which order to reintroduce individual foods, and how often to perform endoscopy and mucosal biopsies for reassessment. More studies on this approach are necessary, including an attempt to evaluate patient QoL given the extensive dietary restrictions often required that involve many “staple” foods. Furthermore, if several foods are to be eliminated simultaneously, enlisting the assistance of a dietitian may be beneficial, particularly in the pediatric population. This may help ensure nutritional requirements are met to facilitate adequate growth and development.

### Pharmacologic management

Current medical therapy for EoE focuses on PPI, corticosteroids and biologics [59, 69].

#### (a) Proton pump inhibitors (PPIs)

PPIs have been shown to have significant and clinically-relevant anti-inflammatory and anti-eosinophil effects that are beneficial in EoE, including inhibiting the expression of intercellular adhesion molecule 1 (ICAM-1) and vascular cell adhesion molecule 1 (VCAM-1) [70], blocking the IL-13 and IL-4 stimulated increase in eotaxin-3 messenger RNA expression and protein secretion [71, 72], and improving epithelial barrier function [73]. In a meta-analysis of 33 studies (total *N* = 619), PPI therapy was associated with clinical and histological remission rates of 61% and 51%, respectively, however, there was a wide range in response rates (23–83%) given the heterogeneity of study designs and populations [74]. A subsequent multicenter observational study found PPI clinical and histological response rates to be comparable at 71% and 49%, respectively, with a lower response rate in patients with preexisting strictures [75]. Given their favourable safety profile and ease of use, PPIs could be considered a reasonable first-line therapeutic option [74]. For patients that fail to respond to a PPI trial, dietary or other medical therapies are often successful.

#### (b) Corticosteroids

Systemic (oral) corticosteroids were one of the first treatment options shown to be effective in patients with EoE. Both clinical and histologic improvement have been noted in approximately 95% of EoE patients using systemic corticosteroids; however, upon discontinuation of therapy, 90% of patients experience a recurrence in symptoms [76]. Furthermore, given that prolonged use of systemic corticosteroids is associated with well-known and potentially serious adverse effects, their long-term use is not recommended. Systemic corticosteroids should be reserved for emergent cases, such as patients with dysphagia requiring hospitalization or patients experiencing significant weight loss or dehydration due to swallowing difficulties.

Given their substantially better safety profile, topical corticosteroids delivered to the esophagus have become the mainstay of pharmacotherapy for patients with EoE. Swallowed fluticasone propionate (500–1000 µg/day), oral viscous budesonide (1000–2000 µg/day), and budesonide in an orodispersible tablet (1000–2000 µg/day) have been shown to be effective in the management of EoE [1, 2, 77, 78]. Fluticasone propionate is delivered via a pressurized metered dose inhaler (pMDI) that is activated into the mouth (without inhaling and without a spacer device) and swallowed. To create oral viscous budesonide, the contents of a vial used for nebulization are mixed with a thickening agent to increase the viscosity of the solution to slow its transit over the esophageal lining and swallowed [30]. A variety of sweeteners/vehicles can be chosen for increasing viscosity, including sucralose, applesauce, and honey [79]. As oral viscous budesonide provides significantly higher medication contact time than nebulized budesonide, only the oral viscous form is recommended [80]. Budesonide in an orodispersible tablet formulation has been approved by Health Canada for adults with EoE [77, 81]. The tablet is placed on the tip of the tongue and pressed gently against the hard palate until it completely disintegrates. For all topical corticosteroids, eating and drinking must be avoided for 30 min after administration.

Randomized clinical trials of topical corticosteroids have shown both histologic and symptomatic improvements in 50–90% of pediatric and adult patients with EoE [77, 78, 80, 82–91]. The most frequent complications noted are oropharyngeal and esophageal candidiasis, in approximately 10% of patients [78]. Some studies have described the potential for adrenal suppression [92–94], although a meta-analysis suggested minimal adverse effects and no evidence of adrenal suppression [91].

After 6–8 weeks of topical therapy, patients should undergo repeat endoscopic assessment to ensure histologic response to therapy. If a therapeutic response is confirmed, treatment should be reduced to the lowest effective dose with appropriate follow up. It is important to note that symptoms and pathological changes often recur after discontinuation of topical corticosteroids. Therefore, many patients with EoE will require long-term treatment.

#### (C) Biologics

As IL-4, IL-5, IL-13 and IgE appear to play a role in the pathogenesis of EoE, humanized monoclonal antibodies against IL-4 receptor-α component (dupilumab), IL-5 (reslizumab, mepolizumab, benralizumab), IL-13 (cendakimab), Siglec-8 (lirentelimab), and IgE (omalizumab) have been proposed as potential therapeutic options for the disease [95, 96].

Dupilumab has demonstrated the most robust evidence to date, and is the only biologic currently approved for the treatment of EoE [97, 98]. Dupilumab is a fully humanized monoclonal antibody that binds to the α-subunit of the IL-4 receptor, inhibiting IL-4 and IL-13 signaling. Dupilumab 300 mg subcutaneously weekly has been found to induce histologic remission in approximately 60% of patients [97]. Dupilumab is currently approved by Health Canada for patients ≥1 year of age with EoE weighing at least 15 kg [99], however, its high cost remains an ongoing concern. Therefore, dupilumab is currently largely reserved for patients with refractory disease, or with other comorbid atopic disease for which it has demonstrated efficacy (atopic dermatitis, eosinophilic asthma or chronic rhinosinusitis with nasal polyposis) so that benefit can be achieved for multiple concurrent atopic conditions [100, 101]. Common adverse reactions with dupilumab include injection-site reactions/pain, upper respiratory tract infection and conjunctivitis [98, 102, 103].

Several other biologics have been studied for the treatment of EoE, with mixed results. Cendakimab (anti-IL-13) has promising preliminary results and is undergoing an open-label phase 3 trial [104, 105]. Results from clinical trials of anti-IL-5 agents (reslizumab, mepolizumab, and benralizumab) and the anti-Siglec-8 agent, lirentelimab, have shown improvement on biopsy but persistence of symptoms [106–108]. Omalizumab (anti-IgE) has not been demonstrated to be effective [109, 110]. None of these biologics are currently approved for the treatment of EoE.

### Endoscopic dilation

Esophageal endoscopic dilation is most commonly used in adults with established esophageal strictures. Although many physicians are fearful to dilate EoE patients due to concerns regarding mucosal tears and perforations, numerous case series attest to the safety and efficacy of esophageal dilation [111], with many patients experiencing symptom relief for an average of 2 years. Furthermore, mucosal tears are in fact a sign of successful dilation, not complications. Periodic dilation is now considered an acceptable alternative to medical or dietary therapy in some healthy adults with EoE [2, 111].

## Prognosis

The long-term prognosis for patients with EoE is unknown. Some patients may follow a “waxing and waning” course characterized by symptomatic episodes followed by periods of remission. There have also been reports of apparent spontaneous disease remission in some patients; however, the risk of recurrence in these patients is unknown. It is possible that long-standing, untreated disease may result in esophageal remodeling, leading to strictures, Schatzki ring and, eventually, achalasia. Progressive remodeling appears to be gradual, but not universal. Also, the duration of untreated disease appears to be the best predictor of stricture risk [4].

To date, neither dietary elimination nor medical therapy has been shown to modify the natural history of EoE [112]. Therefore, maintenance therapy and/or periodic esophageal dilation are important considerations given that the majority of patients with this disease will develop recurrent symptoms and esophageal eosinophilia upon cessation of medical or dietary therapy. Although the natural history suggests that EoE is a chronic, recurrent disease [113], it appears benign with no associated risk of malignancy [112]. More studies are needed to better understand the natural history of EoE.

A recent study found that a gap of care of two years or more leads to increased disease activity and progression to fibrostenosis [114]. Given the chronic nature of this disease and risk of disease progression, individualized clinical follow-up is recommended, and follow-up is recommended at least every 12–24 months, even in patients with stable disease [114, 115].

## Conclusions

EoE is an evolving condition requiring further study to better understand the mechanisms of disease development and tissue injury, natural history, and optimal management. Although clearly an atopic condition, our ability to identify specific allergic triggers remains limited, and this is an important focus of ongoing investigation. As our understanding surrounding EoE improves, so will strategies for the diagnosis and treatment of the condition.

## Data Availability

Data sharing not applicable to this article as no datasets were generated or analyzed during the development of this review.

## Declarations

### About this supplement

This article has been published as part of Allergy, Asthma & Clinical Immunology, Volume 20 Supplement 03, 2024: Practical Guide for Allergy and Immunology in Canada 2024. The full contents of the supplement are available at https://aacijournal.biomedcentral.com/articles/supplements/volume-20-supplement-3

## Abbreviations

EoE: eosinophilic esophagitis
FED: food elimination diet
GERD: gastroesophageal reflux disease
HPF: high power field
ICAM-1: intercellular adhesion molecule 1
IgE: immunoglobulin E
IL: interleukin
MBP: major basic protein
OIT: oral immunotherapy
pMDI: pressurized metered dose inhaler
PPI: proton pump inhibitors
PPI-REE: proton pump inhibitor-responsive esophageal eosinophilia
QoL: quality of life
RNA: ribonucleic acid
SLIT: sublingual immunotherapy
TGF-β: transforming growth factor-beta
VCAM-1: vascular cell adhesion molecule 1
